# Parenting behaviors of mothers and fathers of young children with intellectual disability evaluated in a natural context

**DOI:** 10.1371/journal.pone.0240320

**Published:** 2020-10-13

**Authors:** Rosa Vilaseca, Magda Rivero, Fina Ferrer, Rosa María Bersabé

**Affiliations:** 1 Department of Cognition, Development and Educational Psychology, University of Barcelona, Barcelona, Spain; 2 Municipal Institute of Social Services of Barcelona, Barcelona, Spain; 3 Department of Methodology for the Behavioral Sciences, University of Malaga, Málaga, Spain; King Fahd University of Petroleum & Minerals, SAUDI ARABIA

## Abstract

The aims of this study were to analyze the interactions of mothers and fathers with their children with intellectual disabilities, focusing on certain parental behaviors previously identified as promoting child development, and to explore the relations between parenting and some sociodemographic variables. A sample of 87 pairs of mothers and fathers of the same children were recruited from Early Intervention Centers. The children (58 male and 29 female) were aged 20–47 months. Most of the families (92%) were from the province of Barcelona (Spain), and the remaining 8% were from the other provinces of Catalonia (Spain). Parenting behaviors, divided into four domains (Affection, Responsiveness, Encouragement, and Teaching) were assessed from self-recorded videotapes, in accordance with the validated Spanish version of the PICCOLO (Parenting Interactions with Children: Checklist of Observations Linked to Outcomes). Parents were administered a sociodemographic questionnaire. The results revealed strong similarities between mothers’ and fathers’ parental behaviors. Mothers and fathers were more likely to engage in affectionate behavior than in teaching behavior. Only maternal teaching presented a significant positive relation to the child’s age. With respect to the child’s gender, no differences were observed in mothers’ parenting. Conversely, fathers scored significantly higher in Responsiveness, Encouragement and Teaching (and had higher total parenting scores) when interacting with boys. The severity of the child’s ID had a statistically significant effect only on fathers’ Teaching, which showed lower mean scores in the severe ID group than in the moderate and mild ID groups. Teaching also presented a significant positive relation to mother’s age, but father’s age was not related to any parenting domain. Mothers with a higher educational level scored significantly higher in Encouragement and Teaching, and the fathers’ educational level was not significantly related to any parenting domain. Mothers’ and fathers’ Teaching, and fathers’ Responsiveness, Encouragement and total parenting scores, presented a significant positive relation to family income. Finally, mothers spent more time in childcare activities than fathers, particularly on workdays. Our main conclusion is that mothers and fathers show very similar strengths and weaknesses when interacting with their children with intellectual disabilities during play.

## Introduction

A responsive home environment that includes good parenting and positive parent-child interactions is predictive of good development outcomes in normally developing children [1–6]. These parent characteristics may also predict better outcomes for children with a disability [7–12].

Parenting behaviors that have been shown to support children’s early development include behaviors in the domains of affection, responsiveness, encouragement and cognitive stimulation or teaching [13–18]. The literature shows that supporting children’s early development is especially critical for children with a clearly established disability such as those with intellectual disabilities (IDs), or for children at risk for poor developmental outcomes [19]. For families with a child with a disability, good parenting and positive parent-child interactions may constitute a real challenge. In comparison with typically developing children, children with IDs may provide less salient cues, be less responsive or have behavioral problems [11]; they may also show less emotional expressiveness, difficulties in joint attention, difficulties in language and communication, and behavioral problems, all of which may hinder the establishment of good interaction patterns [10, 20, 21].

Although there is a large body of literature supporting the relationship between positive parenting and child outcomes in typically developing children [3, 22], fewer studies have examined this association in parents of children with developmental disorders such as intellectual disabilities [for a review, see 23]. Positive parenting of children with disabilities is an important area of research, due to the many stressors that these parents experience [24–26] and their possible impact on parenting. Daily parenting stress habitually has to do with the everyday challenges and demands of providing care for children with serious developmental difficulties such as those with ID. This stress may have a critical impact on the development of parenting and, consequently, on children's psychological and developmental outcomes [27, 28]. Compared with parents of children with typical development (TD), parents of children with ID may adopt more intrusive and negative parenting styles [29–33], which may not aid development. The challenges associated with raising a child with ID are numerous [34, 35] and may affect parental behaviors and, at a later date, children’s outcomes and behavior. Despite these difficulties, studies comparing maternal interactions with a child with disabilities and a child with typical development have shown that mothers try to adapt to the child’s characteristics [36]; when interacting with a child with inhibitory control deficits such as Fragile X Syndrome, they are more directive and less conversational [37]. Mothers with a child with ID show a more directive style than mothers with a TD child, but they can be also more supportive and motivating [38]. However, this directive style does not mean that these parents are any less affectionate or positive [12, 39]. Research has shown that parenting styles vary greatly [10], and a positive view of parenting is vital to empower parents of children with ID and to promote their perception of themselves as effective parents [40]. Furthermore, a positive parent-child relationship may be an important compensatory factor for daily parenting stress in families with children with ID [28].

Raising a child with an ID may affect mothers’ and fathers’ parenting in different ways, although we know that having a child with an ID impacts all family members. Family Systems Theory [41] emphasizes the dynamic and interdependent nature of the family unit, in which the experiences of one member potentially affect the entire system. Mothers, fathers and children are all elements inside this system, with interconnected patterns of actions and relationships [2].

Most of the literature on parenting has been conducted with mothers, but the amount of research including both mothers and fathers, or focusing just on fathers, has increased in recent years [28, 42–49]. These studies have highlighted the role that fathers play in their children’s cognitive, emotional, and social development [50–53]. Studies comparing mothers’ and fathers’ parenting have found both similarities [54] and differences [55]; it seems that fathers are more likely to engage in play activities when interacting with their children, while mothers spend more time in caregiving activities [56]. During play they engage in more rough-and-tumble play than mothers, especially with sons, and that this kind of playfulness is beneficial for the construction of attachments to fathers and the regulation of aggression and other behavioral problems [57–59]. It appears that mothers use more objects and toys and more verbal and didactic play techniques during play than fathers [60]. A recent study [61] found that fathers of daughters were more attentively engaged with their child, sang more to them, and used more analytical language and language related to sadness and the body; in contrast, fathers of sons engaged in more physical play, and used language more focused on achievement. As has been suggested [62, 63], maternal and paternal parental behaviors are linked to the child’s developmental outcomes, implying the existence of a complementary system of parenting, with both commonalities and differences between mothers and fathers. However, far fewer studies have examined parenting behaviors in mothers and fathers of the same child with an ID at very young ages [12, 28, 64; see 43 for a revision] even though early intervention programs are increasingly recognizing the value of engaging fathers in home visits [65, 66]. The needs of fathers of children with ID have received less attention in the literature than those of mothers [42, 43, 67], but there is enough evidence to suggest that paternal involvement in families of children with disabilities can have similarly positive impacts on family wellbeing and child outcomes [68–70]. A literature review of paternal involvement in the presence of a disability [71–73] found it to be linked to increased child cognitive competence and enhanced social skills. Moreover, paternal support in the family has been found to reduce maternal stress in families of children with disabilities [28, 74]. So, father involvement during the early years can lead to positive child and family outcomes in families of children with disabilities such as ID. These findings provide strong justification for the need to continue studying the roles of mothers and fathers of the same child with ID, since we know that the involvement of both parents is a critical ingredient of effective developmental intervention [75–81]. Parenting differences may be addressed in a variety of domains, all of which can contribute to understanding family functioning in the context of a child with disabilities. This paper focuses specifically on the study of parents’ behavior during interactions with their children with ID in a naturalistic home context; however, other variables such as family stress or family wellbeing are equally important in order to understand the complexity of studying families with children with disabilities [43, 82–84].

As we noted above, previous studies of parenting in families of children with ID have focused specifically on mothers. However, some studies conducted with mothers and fathers of children with ID in the 1990s [64, 85] suggested a similarity between mothers’ and fathers’ interactions with their children, even though the mothers were much more involved with their children. These results were later confirmed by more recent studies [42, 70, 86]. Also, the stud by Crnic [43] indicated that both mothers and fathers of children with ID behave similarly with their children to parents of children with typical development, although as the child grows older the father’s involvement in their upbringing decreases.

In relation to father play, a study carried out with fathers of children with ID [87] found it to be associated with more child exploration and symbolic play; in addition, fathers with high emotional availability exhibited more symbolic and less exploratory play than their peers with lower emotional availability.

It is true that few empirical studies have been carried out of mothers’ and fathers’ behavior in families with young children with ID. Nevertheless, it appears that fathers and mothers share some fundamental similarities in their parenting behavior, but that certain behavioral domains present difference. Previous research shows that although fathers may not contribute to caregiving tasks to the same degree, when they do so they are competent care providers [43, 88].

The aim of the present study was thus to explore similarities and differences in the parenting behaviors of mothers and fathers of the same child with an ID in a natural context in Spain.

In relation to the existing literature, our study provides new data on parental interactions with young children with ID in Europe, a context in which public policies for conciliation of work, family and personal life have progressively improved in recent years and have helped to increase the levels of joint participation of men and women in the upbringing and education of their children [89]. Therefore, the study of how mothers and fathers from the same family contribute to child development, and especially in the case of children with developmental disabilities, is a highly relevant topic in Europe today.

We focused on the following research questions:

1. How do mothers and fathers of the same child compare on dimensions of parenting (Affection, Responsiveness, Encouragement and Teaching)?
2. Are the parenting behaviors that mothers and fathers demonstrate with their child with an ID related in any way to family-related demographic variables?

## Methods

### Participants

Our sample was composed of 87 pairs of mothers and fathers of the same children. Almost all families (92%) were from the province of Barcelona (Spain), and the remaining 8% from the other provinces of Catalonia (Spain). They were recruited from Early Intervention Centers (EICs), which cater for children from birth to 6 years of age who have or at risk of developmental delay [90]. The following inclusion criteria were applied: (a) age between 20 and 47 months; and (b) Intellectual Disability (associated or not with another type of disability) diagnosed at least six months before the study.

In Spain, Early Intervention Centers offer a universal, free public service, organized by provinces inside each autonomous community. Each center has autonomy for receiving and evaluating cases, and for intervening when necessary. Access to EICs is by indication or referral by a child care service (health, educational or social services) or by direct contact made by the family. EICs are staffed by professionals from different disciplines (usually neuropaediatricians, psychologists, physiotherapists, speech therapists and social workers). The size of the team is related to the number of population to be served, and the coordinator is usually a psychologist.

The study sample comprised 87 children, 58 male (67%) and 29 female (33%), aged from 20 to 47 months (*M* = 33.0, *SD* = 6.8). Fifty-six per cent of the children were younger than 3 years old (20 to 35 months), and 44% were 3 years old or over (36 to 47 months). The degree of ID was mild (from 33 to 64%) in 46%, moderate (from 65 to 74%) in 45% and severe (> 75%) in 9% (in Spain, the assessment of the percentage of disability is a standardized process carried out by a governmental agency, the Valuation and Guidance Services for People with Disabilities; after diagnosis, the agency issues an official certificate reflecting both the existence and degree of disability, with ID being graded as mild, moderate or severe). These services carried out the assessment and established the degree of disability.

Bayley’s percentile scores were as follows: cognitive development ranged from 0.1 to 84 (*M* = 17.3, *SD* = 18.6); language development from 0.1 to 86 (*M* = 8.7, *SD* = 14.9); and motor development from 0.1 to 76 (*M* = 10.3, *SD* = 15.1). Bayley’s mental age scores were as follows: cognitive development from 0.1 to 42 (*M* = 22.2, *SD* = 8.06); receptive language development from 0.2 to 42 (*M* = 18.3, *SD* = 9.5); expressive language development from 2 to 35 (*M* = 16.3, *SD* = 7.5); fine motor development from 5 to 38 (*M* = 21.5, *SD* = 7.6); and gross motor development from 4 to 38 (*M* = 19.0, *SD* = 6.4).

Mothers were aged 22 to 45 years (*M* = 37.3, *SD* = 4.4). Almost all (98%) were married or living with a partner. Eleven per cent had received only elementary schooling, 40% had completed secondary school, 34% had a university degree, and 15% had post-graduate studies. Most were in full-time (51%) or part-time employment (31%), while 18% cared for their children and were fully responsible for housework.

Fathers were aged 24 to 60 years (*M* = 38.9, *SD* = 5.1). Most (98%) were married or living with a partner. Twenty-two per cent had received only elementary schooling, 36% completed secondary school, 31% had a university degree, and 11% had completed post-graduate studies. Most of them were in full-time employment (91%); the rest were employed part-time (1%), or unemployed (8%). Twenty-three per cent of the families had a monthly income between €1,602 and €2,451, considered an average income in Spain [91]. Monthly income was below €1,602 in 22% of the families and above €2,451 in 44%.

### Instruments

A brief *sociodemographic questionnaire* was used to record the child’s age (in months) and gender, degree of ID (mild, moderate or severe), whether s/he attended a kindergarten or a preschool center, and the parents’ age (in years), gender, marital status, educational level (1: elementary studies, 2: high school, 3: university degree, 4: Master’s/PhD), employment status (full-time employment, part-time employment, unemployed/homemaker), level of monthly income according to the Socioeconomic Classification [92] (from 1: Less than €1,312 to 6: More than €3,005), and daily hours dedicated to child care. In particular, parents were asked: “On average, how many hours a day do you spend on child care (for example, taking your child to school, bathing him/her, taking him/her out for a walk, playing, cooking…) during work? And on the weekend?”

The Spanish version [93] of the Parenting Interactions with Children: Checklist of Observations Linked to Outcomes (PICCOLO) [17, 18] was used to assess parenting. The PICCOLO is a reliable and valid 29-item measure of parent–child interactions for parents with children between the ages of 10 and 47 months. The 29 items reflect parental behaviors linked to children’s developmental outcomes and the measure can be used to assess families with children with disabilities. The PICCOLO items are scored on a 3-point rating scale, from 0 (absent, no behavior observed) to 1 (barely, or some brief, minor, or emerging behavior) to 2 (clearly, definitive, strong or frequent behavior). This rating scale is similar to a behavior checklist, with a yes/no response; a score of 2 corresponds to a clear presence of the behavior and 0 to a clear absence. 1 is an intermediate score, corresponding to behaviors that appear infrequently or not consistently. The items are rated according especially to the consistency of parental behaviors in relation to the child’s actions. For example, in the case of item 3 on the Teaching scale “Repeats or expands child’s words or sounds” a score of 0 reflects the total absence of that behavior and 2 to its consistent presence (when, every time the child utters a sound or a word, the adult consistently repeats or expands it). A score of 1 would be awarded when the adult occasionally performs the behavior but does not respond to many of the child’s utterances (missing opportunities). The advantage of the PICCOLO 3-point scale is that it is easy to score short observations (between 8 and 10 minutes or so) without counting or timing behaviors [17, 18].

As adult-child interaction is a reciprocal process, and as the child’s behavior has an impact on parental behavior, it is important to stress that the PICCOLO items refer to parental behaviors in the context of interaction with the child. The assessment of parenting is based on the child’s behavior during the interaction. Although some items can be assessed by focusing on the adult (for example, “Smiles at the child”; “Talks to the child about characteristics of objects”), most items assess the adult’s behavior in response to that of the child (for example, “Changes pace or activity to meet child’s interests or needs”; “Responds to child’s emotions”; “Replies to child’s words or sounds”; “Verbally encourages child’s efforts”. So, for example, item 3 on Responsiveness (“Is flexible about child’s change of activities or interests”) will score 0 when, every time the child changes his/her focus of interest, the parent is not flexible and does not accept the child’s choice or initiative.

The items are grouped into four domains: (a) Affection (7 items), which involves the physical and verbal expression of affection, positive emotions, positive evaluation and positive regard; (b) Responsiveness (7 items), which includes reacting sensitively to a child’s cues and expressions of needs or interests and reacting positively to his/her behavior; (c) Encouragement (7 items), which considers parents’ support of children’s efforts, exploration, independence, play, choices, creativity, and initiative; and (d) Teaching (8 items), which includes cognitive stimulation, explanations, conversation, joint attention, and shared play. The instrument generates a score for each dimension between 0 to 14 (and 0 to 16 for the Teaching dimension) and a total score between 0 and 58 (adding all the items). The original instrument’s reliability is good [17, 18].

The Spanish version of the PICCOLO was recently validated using a sample of 203 mother–child dyads [93]. The results of the confirmatory factor analysis confirmed that the instrument has a four-factor structure of first order domains (Affection, Responsiveness, Encouragement, and Teaching) that collapses into a single, second-order factor (parenting). It also found a high interrater reliability; the intraclass correlation coefficients (ICC) ranged from .69 for the responsiveness domain to .85 for the total score. With respect to internal consistency reliability, all domain and total scores showed satisfactory Cronbach’s alpha coefficients (.65 for Affection, .75 for Responsiveness, .76 for Encouragement, .72 for Teaching, and .88 for the total score). In this study, Cronbach’s α values for mothers (*N* = 87) and for fathers (*N* = 87) were, respectively: .54 and .55 for Affection; .81 and .84 for Responsiveness; .80 and .84 for Encouragement; .66 and .70 for Teaching; and .88 and .90 for the total PICCOLO score.

### Procedure

Ethical approval was obtained from the Network of Ethics Committees in Universities and Public Research Centers in Spain. Approval was given in accordance with the International Ethical Guidelines for Health-related Research Involving Humans.

Then, several EICs were contacted by letter and telephone and informed of the nature of the study, and the coordinators of the centers were asked to help in recruiting families for the study. Families were informed that their participation would be entirely voluntary and anonymous. They received a letter with information about the study, the sociodemographic questionnaire and a brief guide about how to video-record adult-child interactions during play at home. The parents signed an informed consent document. Mothers and fathers were asked to auto-record, separately, between 8 and 10 minutes of a normal play session with their child at home, with the following instruction “Interact and play with your children as you typically do”. Ninety-four per cent of the videos collected in this sample were more than nine minutes long. The father’s and mother’s recordings could be made on the same day or on different days, within a maximum period of one week. Both parents chose what to play with their child. Some games and materials were suggested in the brief guide: for example, books, toy animals, kitchens, little dolls, or building blocks. Finally, the videos were collected and scored according to the PICCOLO criteria. Only videotapes that complied with the researcher’s instructions (99%) were scored.

We opted for video recording and self-recording because video is considered advantageous to live observation and live coding as it permits researchers to pause and replay the activity as often as is necessary for a thorough analysis of the data [94], and self-recording avoids the interference of the presence of a third person. Parents were aware of the overall aims of the research, but they did not know which specific behaviors were being analyzed.

Finally, the videos were collected and scored according to the PICCOLO criteria by a small group of psychologists and specialists in child development. The first author of this paper, who had been trained by the authors of the PICCOLO, trained the group of raters for this study. The trainees read the PICCOLO manual and watched and discussed the scores for four video recordings with the expert coder. After the training sessions, each observer scored four to six additional video recordings in order to establish reliability prior to collecting study data. Observers were considered to have completed their training when they presented an inter-rater agreement of 80% or more with the expert coder, following the same criteria as the PICCOLO user’s guide [18]. Each coder scored roughly 20 video recordings selected randomly, including both mothers and fathers from the same different or families. Only videotapes that complied with the researcher’s instructions were scored (however, 99% were deemed satisfactory).

### Data analysis

Differences in mean PICCOLO item scores between mothers and fathers of the same child were compared via the Wilcoxon signed-ranks test for paired samples, while their differences on mean domain and total PICCOLO scores were compared via Student’s *t*-test for paired samples. Effect size was calculated using Cohen’s *d*. The relationship between mothers’ and fathers’ parenting scores was analyzed by computing Pearson’s correlation coefficients.

For categorical sociodemographic variables, mean parenting scores were compared via Student’s *t*-test (for comparing two means) or via robust Brown-Forsythe ANOVA (for more than two means). The relationship between parenting scores and demographic variables was examined via Pearson product-moment correlation coefficients (for continuous variables), or via Spearman correlation coefficients (for ordinal variables). Missing data were handled by pairwise deletion. IBM SPSS (version 24.0 for Windows) was used for all the statistical analyses.

## Results

### Mothers’ and fathers’ parenting

Table 1 presents descriptive statistics (mean and standard deviations) of PICCOLO item scores for mothers and fathers separately. One of the items in the Affection domain (item 5), one in the Encouragement domain (item 6), and five in the Teaching domain (items 1, 2, 5, 6 and 7) showed mean scores lower than 1, in both mothers and fathers, which indicates that the corresponding behavior was barely observed in either parent. The order of the three items with the highest mean was the same for both mothers and fathers: two items in the Affection domain (“Speaks in a warm tone of voice”, “Is physically close to child”) and one item in the Responsiveness domain (“Pays attention to what child is doing”). This indicates that these parenting behaviors were the ones most clearly observed in the majority of mothers and fathers.

**Table 1:** Descriptives of PICCOLO item scores for mothers and fathers.

Domains and Items	Mothers (*n* = 87)		Fathers (*n* = 87)		*Wilcoxon*
	*M*	*SD*	*M*	*SD*	*Z*
*Affection*					
1. Speaks in a warm tone of voice	1.94	.27	1.87	.36	1.73
2. Smiles at child	1.68	.53	1.52	.67	2.01*
3. Praises child	1.59	.60	1.53	.66	0.65
4. Is physically close to child	1.84	.45	1.85	.41	-0.21
5. Uses positive expressions with child	0.67	.88	0.49	.75	1.41
6. Is engaged in interacting with child	1.72	.52	1.64	.55	1.12
7. Shows emotional warmth	1.78	.41	1.65	.54	2.07*
*Responsiveness*					
1. Pays attention to what child is doing	1.77	.44	1.67	.56	1.70
2. Changes pace or activity to meet child’s interests or needs	1.25	.79	1.19	.81	0.54
3. Is flexible about child’s change of activities or interests	1.19	.80	1.08	.82	1.01
4. Follows what child is trying to do	1.61	.57	1.58	.62	0.54
5. Responds to child’s emotions	1.44	.72	1.38	.70	0.74
6. Looks at child when child talks or makes sounds	1.60	.55	1.55	.62	0.84
7. Replies to child’s words or sounds	1.35	.75	1.41	.75	-0.85
*Encouragement*					
1. Waits for child’s response after making a suggestion	1.24	.75	1.27	.82	-0.46
2. Encourages child to handle toys	1.69	.55	1.51	.60	2.57*
3. Supports child in making choices	1.18	.75	1.09	.81	0.81
4. Supports child in doing things on his/her own	1.31	.71	1.31	.68	-0.23
5. Verbally encourages child’s efforts	1.43	.72	1.35	.67	0.86
6. Offers suggestions to help child	0.94	.79	0.93	.74	-0.13
7. Shows enthusiasm about what child is doing	1.60	.59	1.47	.67	1.62
*Teaching*					
1. Explains reasons for something to child	0.27	.56	0.16	.45	1.71
2. Suggests activities to extend what child is doing	0.89	.83	0.97	.83	-0.97
3. Repeats or expands child’s words or sounds	1.16	.82	1.09	.82	0.87
4. Labels objects or actions for child	1.60	.59	1.42	.70	2.17*
5. Engages in pretend play with child	0.69	.86	0.48	.78	1.86
6. Does activities in a sequence of steps	0.48	.71	0.49	.74	-0.23
7. Talks to child about characteristics of objects	0.98	.83	0.80	.79	1.66
8. Asks child for information	1.43	.70	1.31	.73	1.27

Note:

* p < .05

Differences in mean PICCOLO item scores between mothers and fathers of the same child were compared via the Wilcoxon signed-ranks test for paired samples. Mothers showed higher mean scores than fathers (*p* < .05) in two items from the Affection domain, one from the Encouragement domain, and one from the Teaching domain (see Table 1). This result means that positive parental behaviors corresponding to those items were more frequently observed in mothers than fathers.

Table 2 presents descriptive statistics (mean and standard deviations) of PICCOLO domains and total scores for mothers and fathers. Scores were computed as means, i.e., dividing the sum score by the number of items in each domain. Thus, mean scores for all domains ranged theoretically from 0 to 2, like the item scores, so they have a common interpretation regardless of the number of items they contain. For both sets of parents in this study, all mean scores ranged between 1 (bare, brief, minor, or emerging behaviors were observed) and 2 (clear, definite, strong, or frequent behaviors were observed), except for the Teaching domain, which showed a mean lower than 1. In other words, both mothers and fathers tend to show positive parenting behaviors (Affection, Responsiveness, Encouragement) with their children, except for teaching behaviors, which were rarely observed.

**Table 2:** Differences between mothers and fathers on PICCOLO domains and total scores (N = 87).

PICCOLO score	Mothers		Fathers		*t*_(86)_	Cohen’s *d*
	*M*	*SD*	*M*	*SD*		
*Affection*	1.60	0.28	1.51	0.30	2.44*	0.26
*Responsiveness*	1.45	0.46	1.41	0.49	0.95	0.10
*Encouragement*	1.33	0.47	1.28	0.51	0.93	0.10
*Teaching*	0.93	0.40	0.84	0.42	1.99*	0.21
*Total*	1.31	0.32	1.24	0.35	1.86	0.20

Note:

* *p* ≤ .05

Differences in means on each domain and the total PICCOLO scores between mothers and fathers of the same child were compared via Student’s *t*-test for paired samples. As also shown in Table 2, mothers showed higher mean Affection and Teaching domain scores than fathers. However, the effect size of the differences between mothers’ and fathers’ mean Affection and Teaching scores can be considered as small (*d* ≈ .20), using Cohen’s benchmarks for interpreting effect sizes [95].

**Fig 1:**
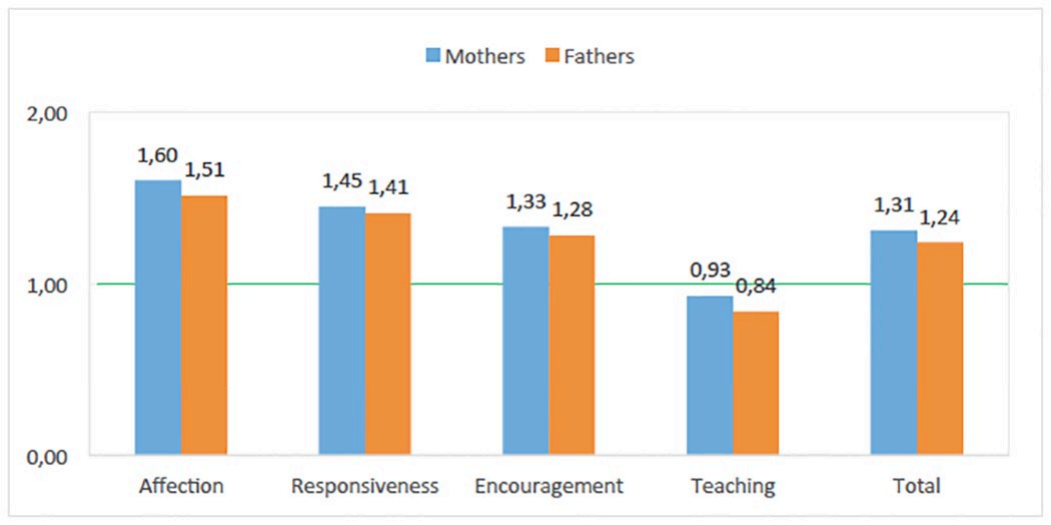
PICCOLO domain and total mean scores for mothers and fathers.

PICCOLO mean scores are represented graphically in Fig 1. As can be observed, mean scores on the four positive parenting domains followed a similar pattern in mothers and fathers: that is, the order of the mean scores was the same. For both parents, the highest mean score corresponded to the Affection domain, followed by the Responsiveness and Encouragement domains; the lowest mean scores were on the Teaching domain (*M* < 1).

The relationship between mothers’ and fathers’ parenting scores was analyzed by computing Pearson’s correlation, which showed a statistically significant positive correlation between parents on Affection (*r* = .313; *p* = .003), Responsiveness (*r* = .501; *p* < .001), Encouragement (*r* = .403; *p* < .001), Teaching (*r* = .492; *p* < .001) and the total PICCOLO score (*r* = .458; *p* < .001). This result indicates the relative similarity between mothers and fathers on all parenting domains.

### Sociodemographic variables and parenting

The associations between positive parenting and each of the variables included in the sociodemographic questionnaire were analyzed. Pearson’s correlation coefficients between child age (in months) and mothers’ and fathers’ PICCOLO scores were computed. The only statistically significant positive correlation was found between child age and scores for the Teaching domain, in mothers (*r* = .266; *p* = .013), indicating that mothers’ teaching behaviors were more frequently observed with older children. In contrast, none of the fathers’ parenting domains was significantly related to child age.

With respect to child gender, the Student’s *t*-test for independent samples found no statistically significant differences between boys (*n* = 58) and girls (*n* = 29) on PICCOLO mean domain and total scores, for mothers (*p* < .05). This result shows that positive parenting interactions observed in the mothers were not related to child gender. However, the positive parenting interactions observed in the fathers (except for the Affection domain) were more frequently observed for boys than girls.

The difference in the fathers’ mean Responsiveness score between boys (*M* = 1.51; *SD* = 0.46) and girls (*M* = 1.21; *SD* = 0.52), was statistically significant (*t*(85) = 2.72; *p* = .008). The fathers’ mean Encouragement score was also higher for boys (*M* = 1.39; *SD* = 0.48) than for girls (*M* = 1.08; *SD* = 0.52) (*t*(85) = 2.73; *p* = .008), as was their mean Teaching domain (boys *M* = 0.94; *SD* = 0.41, girls *M* = 0.66; *SD* = 0.38) (*t*(85) = 3.02; *p* = .003). Fathers’ total PICCOLO scores were also higher for boys (*M* = 1.32; *SD* = 0.34) than for girls (*M* = 1.09; *SD* = 0.33) (*t*(85) = 2.97; *p* = .004).

Comparison of parenting scores between the three child ID severity groups (mild, moderate or severe) was analyzed via Brown-Forsythe ANOVA. The results indicated that none of the mothers’ mean scores on the PICCOLO domains and total scores differed significantly (*p* > .05) between the three severity groups. Similarly, fathers’ mean PICCOLO scores did not differ across the three groups, except for the Teaching domain. The first group of fathers with a child with a mild ID (*N* = 40) obtained mean Teaching scores of 0.94 (*SD* = 0.44); the mean for the second group (moderate ID, *N* = 39) was 0.83 (*SD* = 0.38); and the mean for the third group (severe ID, *N* = 8) was 0.44 (*SD* = 0.28). ANOVA results showed statistically significant differences in mean Teaching scores in at least two ID severity groups (*F*(2, 84) = 5.34; *p* = .007). Pairwise comparisons analyzed via Tukey’s HSD test showed that the mean Teaching scores of the group with severe ID differed significantly from those of the mild or moderate ID groups (*p* < .05). However, no differences were found between the means for the mild and moderate ID groups (*p* > .05).

Several parents’ sociodemographic characteristics were also taken into account in the analysis. With respect to mothers’ age, a statistically significant correlation was found with Teaching domain scores (*r* = .264; *p* = .013), in that teaching behaviors were more frequently observed in older mothers. However, fathers’ age was not significantly related (*p* > .05) to any of the parenting domains.

In relation to parents’ educational level (from 1: Elementary studies to 4: Master’s/PhD), statistically significant Spearman correlation coefficients were found for mothers on the Encouragement (*r*_*s*_ = .218; *p* = .042) and Teaching (*r*_*s*_ = .339; *p* = .001) domain scores and the total PICCOLO scores (*r*_*s*_ = .280; *p* = .009). Thus, mothers with higher educational levels performed more positive parenting behaviors (except for the Affection and Responsiveness domains) in their interactions with their children. However, fathers’ educational level was not significantly related to their positive parenting behaviors.

Mean parenting scores were compared across three groups of mothers’ working status via robust Brown-Forsythe ANOVA, for more than two independent means. Differences between the three groups of mothers on mean parenting scores were not statistically significant (*p* > .05). Most of the fathers were in full-time employment (91%) and thus the sample size of the partially employed (*n* = 1) and unemployed/homemakers (*n* = 7) was too small to allow an analysis of the relationship between fathers’ employment and parenting.

Marital/partner status was also included in the sociodemographic questionnaire but it was not analyzed, as almost all parents in the study sample were married or living with a partner (98%) and almost all of these couples (97%) were parents of the same child. With respect to family income (from 1: lower than €1,602, to 6: higher than €2,451), statistically significant Spearman correlation coefficients were found between family income and mothers’ Teaching (*r*_*s*_ = .297; *p* = .006), fathers’ Responsiveness (*r*_*s*_ = .271; *p* = .013), fathers’ Encouragement (*r*_*s*_ = .270; *p* = .013), fathers’ Teaching (*r*_*s*_ = .233; *p* = .033), and fathers’ total PICCOLO scores (*r*_*s*_ = .264; *p* = .015). Therefore, various aspects of positive parenting are related to family income, to a greater extent in fathers than in mothers.

Finally, with respect to the amount of time that parents dedicate to childcare, the results showed that, on workdays, mothers devoted more time (*M* = 7.56; *SD* = 3.22) than fathers (*M* = 4.99; *SD* = 3.16). The *t*-test for paired samples indicated that the difference between means was statistically significant (*t*_(79)_ = 5.91; *p* < .001) with a large effect size (Cohen’s *d* = .66). On the weekend, mothers also spent more hours carrying out childcare activities (*M* = 11.40; *SD* = 2.20) than fathers (*M* = 10.55; *SD* = 2.77), as shown by a statistically significant difference (*t*_(79)_ = 2.74; *p* = .007) with a small effect size (Cohen’s *d* = .31).

## Discussion

This study aimed to contribute to the understanding of parenting constructs across gender by including the same measure for mothers and fathers. Our first aim was to explore whether mothers and fathers of children with an Intellectual Disability (ID) differed in terms of parenting dimensions when they were evaluated playing in a natural context at home. Comparative analyses showed small differences between mothers and fathers of the same child. Even though mothers scored higher than fathers on most PICCOLO items, only four items showed statistically significant differences. Previous evidence using the same measure indicated that mothers usually score higher than fathers [96–99]. What is particularly interesting from our point of view is that the items with the highest lowest means were the same for both mothers and fathers and that the order of the PICCOLO subscale scores, from highest to lowest, was also the same. The coincidence of high scores on the same parenting domain scores in mothers and fathers suggest that their patterns of parenting are similar. Previous studies of mothers and fathers of children with normative development suggests that their parenting behaviors are conceptually very similar [100–104]; equally, our data from mothers and fathers with young children with ID suggest that there are no separate dimensions of fathering and mothering. Those results are similar to those recorded in the studies carried out with mothers and fathers of children with ID mentioned in the introduction section [28, 43, 64, 85].

In relation to the PICCOLO’s distinct domains, mothers and fathers of children with disabilities engage in more types of affective behavior (closeness, warmth…) and fewer teaching behaviors (conversation, play, language and cognitive stimulation…). We should bear in mind that this study included very young children, with cognitive and language levels below their chronological age, so these parental behaviors are probably very similar to those shown by fathers and mothers towards much younger children without disabilities [17, 105]. The Affection and Teaching domains showed statistically significant differences between mothers and fathers but the effect size of these differences was small. Teaching involves activities that include cognitive stimulation, shared conversation and play, explanations, labeling and joint attention, which are essential for promoting child development [14, 106, 107]. However, in children with IDs these activities may be difficult to carry out. As mentioned in the introduction, children with IDs may have problems with joint attention and with language and communication, which may contribute to the difficulties establishing good interaction patterns [21]. This is something that early intervention practitioners should take into account.

Most EICs in Spain focus on a child-centered approach, mainly aimed at the rehabilitation of the problems or difficulties of children and their families. Professionals at these centers intervene with the child with disabilities far from their natural environment. Recently, there has been growing interest among professionals in increasing family participation in early intervention as a way to improve early childhood intervention outcomes [108, 109]. We strongly believe that the information on behaviors identified by the PICCOLO could help guide professionals’ intervention plans, and also that the Teaching domain is one of the aspects that should be emphasized in young children with IDs and their families when following family capacity-building practices [110]. The PICCOLO has been used with children with identified disabilities such as ID or Autism Spectrum Disorder in large samples in the US [11], Spain [12], Saudi Arabia [111] and Germany and Switzerland [36]. These studies have demonstrated this checklist’s strong reliability and predictive validity for families with children with a disability; indeed, in two of these studies the early parenting behaviors measured with the PICCOLO predicted cognitive and language outcomes in children with disabilities [11, 12]. This underlines the importance of early parenting in children with disabilities, as has also been emphasized by family-centered models in the field of early intervention [112–114]. Early intervention professionals should support parents in interactions with their children in natural routines through collaboration with families to promote functional learning and optimal outcomes [115]. Coaching with the PICCOLO increases positive parent-child interaction [116]. When early intervention professionals give feedback to parents about their parenting, both parent skills and child outcomes improve [117, 118].

The second aim of this study was to explore the relationship between mothers’ and fathers’ parenting and family-related demographic variables. Our findings in relation to child age and mothers’ and fathers’ PICCOLO scores only showed a statistically significant positive correlation between child age and scores for the Teaching domain, in mothers, meaning that mothers’ teaching behaviors were more frequently observed with older children. These results corroborate those of earlier research on mothers in the general population [17] and mothers with children with a disability [11]. However, none of the fathers’ parenting domains was significantly related to child age. This means that parents’ parenting behavior does not depend so much on the child's age but on the type of activity in which they engage [63]. Our results did not show an association between parenting interactions in Spanish mothers and child gender, but Spanish fathers presented more positive parenting interactions (except for the Affection domain) with boys than with girls. Some studies in families with children of normal development have shown different parenting styles for sons and daughters [119, 120]; few studies have examined this difference in mothers and fathers of children with an ID. A relevant finding was that the mothers’ parental behaviors did not vary according to the child’s degree of disability. Almost the same could be said of fathers, with the exception of the Teaching domain, in which fathers of children with severe ID showed significantly lower scores. We believe that engaging in Teaching interactions is particularly difficult for these fathers, because it tends to be the mothers who attend the early Intervention Centers, and the service providers are more likely to provide mothers with advice regarding behaviors in the Teaching domain (for example, “Labels objects or actions to the child”; “Repeats or expands child’s words or sounds”; or “Asks child for information”). Father’s teaching is a very important parenting domain; previous research by our group has shown that it is related to cognitive development in children with ID [12]. These results suggest that specific strategies should be developed to involve fathers in this parenting domain in the intervention at the EIC. As noted above and also in previous research [121, 122] EI service providers may have limited understanding of effective strategies for involving fathers, because mothers continue to be the main participants at these centers. Regarding other parental factors, the research suggests that parental age is related to parenting behaviors, in the sense that older parents seem to be more likely to raise children with greater emotional stability [123]. Our results showed that fathers’ age was not significantly related to any of the parenting domains but that mothers’ age was correlated with Teaching domain scores, thus corroborating the results of a similar study [124]. However, more longitudinal research is needed to validate these results, especially in older fathers and mothers of children with disabilities. Regarding level of schooling, our results showed that mothers with higher educational levels showed more positive parenting behaviors in their interactions with their children than those with lower levels. However, a higher level of schooling among fathers was not significantly related to positive parenting behaviors. These findings are at odds with those of previous reports [125, 126]: they may be due in part to role specialization, whereby mothers are more likely to be engaged in childcare [127] or there may be other factors such as family income which, as our data have shown, are significant. Finally, with respect to the amount of time that parents dedicate to childcare, our results show that mothers spend more time carrying out childcare activities than fathers, although this difference is more evident on weekdays than on weekends. We know that fathers today spend more time with their children [128], but the amount of time that parents spend with their children depends above all on whether they work inside or outside the home. In our sample, there was a major difference between mothers and fathers in this variable: 91% of fathers work outside the home, while 18% of mothers did not work and 31% worked only part-time in order to be able to take care of their children. These results confirm the findings of previous studies regarding the influence of the employment status of parents of children with IDs [74, 129], and may account for the differences between mothers and fathers. It is a fact in Spain that when a child with a disability is born in a family, it is usually the mother who stops working to take care of the child. But this does not necessarily mean that father-child interactions are less positive; fathers can compensate for a smaller quantity of time by spending higher “quality” time with their children [54]. We are convinced of the importance of including fathers in research and in intervention programs, and in fact this study confirms the findings of previous studies regarding the similarity in the dimensions of mothers’ and fathers’ parenting [63, 100, 103]. In Spain, as in other countries [68, 130, 131], fathers are noticeably absent in EICs This study highlights the need for paternal involvement in EI, since we know that fathers, jointly with their partners, have a positive impact on the development of children with disabilities [68, 75]. Few studies have examined the measurement equivalence of parenting dimensions in mothers and fathers with a child with ID. This is necessary if we want to have a full picture of parenting and its influence on the development of children, especially in families with children with ID.

### Limitations and future directions for research

This study has several limitations that should be taken into consideration. The first is the selection of the sample. Although participants were recruited at Early Intervention Centers through contact with the center coordinators, the procedure used to select the participants may have been conditioned by the willingness of families to participate [132]; probably, the parents who took part were the ones who were most knowledgeable about child development, and most aware of the importance of parental interactions, or even the most confident about their parenting skills. Similarly, it may be that the parents who were more worried about their child’s development were reluctant to participate. Second, this is a descriptive study and there were no observational measures of parenting over time. Mothers’ and fathers’ patterns of parenting behavior may change as time passes depending on factors such as the age of the child, family structure, socioeconomic status and employment [43, 54, 129]. Neither the behavior of the child, nor its influence on the adult’s behavior, was directly analyzed. Although the PICCOLO items refer to parental behaviors in the context of adult-child interaction, and therefore reflect to a certain extent the adult’s responses to the child’s behavior, future studies might incorporate a dyadic analysis that includes a coding of the observed child’s behavior. In addition, in this study we did not analyze whether the parental behaviors of mothers and fathers predict children’s subsequent cognitive and linguistic development. The relation between parenting and child development needs to be explored, as we stress in an earlier study by our group [12]. Nor did we include measures of the quality of mothers’ and fathers’ parenting behaviors especially if both parents live together (as they did in practically all our cases). Although we asked about the amount of time they spent with their sons and daughters but we did not ask about the types of activities they engaged in. In future studies the interactive effects of mothers’ and fathers’ parenting behavior on children should be analyzed. This is a very important issue, especially in families with children with an ID, but it is something that is difficult to assess. Further, our sample size was modest, and so our findings require replication before we can draw conclusions about the similarities and differences of parenting behaviors in mothers and fathers of the same child with ID. Finally, we stress that this study is based on a non-experimental (correlational or quasi-experimental) methodology which is able to suggest the existence of relationships, but cannot establish the direction of the causality of the relationships it identifies.

## Supporting information

S1 Data (DOCX)
