# Supplementary material for: Parenting behaviors of mothers and fathers of young children with intellectual disability evaluated in a natural context
Source: PLoS One. 2020 Oct 13;15(10):e0240320. doi: 10.1371/journal.pone.0240320 (PMC7553331; doi:10.1371/journal.pone.0240320)
Supplement: S1 Data — (DOCX) [file pone.0240320.s001.docx]

**S1 Data.**

[**https://doi.org/10.6084/m9.figshare.12501398.v4**](https://doi.org/10.6084/m9.figshare.12501398.v4)

**(SAV)**
